# Effectiveness of a Motivational Interviewing-Based Intervention in Decreasing Risky Alcohol Use in Primary Care Patients in Spain: A Controlled Clinical Trial

**DOI:** 10.3390/healthcare12191970

**Published:** 2024-10-02

**Authors:** Celia Pérula-Jiménez, Esperanza Romero-Rodríguez, José Angel Fernández-García, Juan Manuel Parras-Rejano, Ana Belén Carmona-Casado, Manuel Rich-Ruiz, Ana González-De la Rubia, Juan Baleato-Gomez, on behalf of the Collaborative Group ALCO-AP20 Study

**Affiliations:** 1 Pedro Abad Health Center, UGC Montoro, Andalusian Health Service, 14630 Cordoba, Spain; celia.perula.sspa@juntadeandalucia.es; 2 Maimonides Biomedical Research Institute of Cordoba (IMIBIC), Reina Sofía University Hospital, University of Córdoba, 14004 Cordoba, Spain; jangelfernandezgarcia@gmail.com (J.A.F.-G.); juanm.parras.sspa@juntadeandalucia.es (J.M.P.-R.); anabelen.carmona@imibic.org (A.B.C.-C.); en1rirum@uco.es (M.R.-R.); 3 Carlos Castilla del Pino Health Center, Andalusian Health Service, 14011 Cordoba, Spain; 4 Córdoba Guadalquivir Health District, Andalusian Health Service, 14011 Cordoba, Spain; ana.gonzalez.rubia.sspa@juntadeandalucia.es; 5 Villarrubia Health Center, UGC Occidente-Azahara, Andalusian Health Service, 14005 Cordoba, Spain; 6 Huerta de la Reina Health Center, Andalusian Health Service, 14600 Cordoba, Spain; 7 PAPPS Evaluation and Improvement Group (semFYC), 08009 Barcelona, Spain; 8 Faculty of Medicine and Nursing, University of Córdoba, 14004 Cordoba, Spain; 9 General Emergencies Unit, Regional University Hospital, 29010 Malaga, Spain; juan.baleato.sspa@juntadeandalucia.es

**Keywords:** motivational interviewing, primary care, risky alcohol use, clinical trial

## Abstract

Objective: Our study aimed to evaluate the effectiveness of an intervention based on Motivational Interviewing (MI) performed by healthcare professionals in Primary Care (PC) patients with risky alcohol use through a multicenter, two-arm parallel, cluster-randomized, open-label controlled clinical trial. Methods: PC professionals were randomized into two groups: an Experimental Group (EG) and a Control Group (CG). The study was carried out in PC centers of the Andalusian Health Service, located in Cordoba, Spain. An MI-based approach was implemented with patients recruited in the EG, while health advice was provided to those included in the CG. The follow-up period was 12 months, with five visits scheduled. The consumption of standard drinking units per week was quantified, and risky alcohol use was estimated using the Alcohol Use Disorders Identification Test (AUDIT). An intention-to-treat statistical analysis was performed. Relative risk (RR), absolute risk reduction (ARR) and the number of subjects needed to treat (NNT) were used to estimate the intervention effect size. Results: A total of 268 patients were included, 148 in the EG and 120 in the CG. Considering the quantification of risky alcohol use, the ARR at 12 months after baseline visit was 16.46% (95% CI: 5.37–27.99), with an NNT of 6 (95% CI: 4–19). According to the AUDIT, the ARR at 12 months was 13.15% (95% CI: 2.73–24.24%), and the NNT was 8 (95% CI: 4–37). Conclusions: We concluded that MI is more effective than the usual health advice in decreasing risky alcohol use in patients treated in PC.

## 1. Introduction

Alcohol consumption is the main risk factor for developing chronic diseases in the population aged 15 to 49 years worldwide [1]. The World Health Organization (WHO) reports that 5.1 per cent of the global burden of disease and injury is attributable to alcohol use, calculated in terms of disability-adjusted life years (DALYs) [2]. In the same vein, according to the study of the global burden of disease (GBD, 2019), alcohol use is the main risk factor in terms of DALYs worldwide for people aged 25 to 49 years [3]. It is a causal factor in more than 200 diseases and disorders [4,5], and it was one of the factors responsible for the 92,000 deaths from cancer in the WHO European Region in 2018 [6].

According to global data, alcohol is the most used psychoactive substance by the population in Spain, and the prevalence of alcohol consumption in the last month was 64.5% [7]. It is estimated that 4.2% of the Spanish population could present risky alcohol use, and around 15,489 Alcohol Attributable Deaths (AADs) occurred annually in residents in Spain aged ≥ 15 years during the period 2010–2017 [8]. In 2019, 9964 episodes related to alcohol poisoning were reported in hospital emergency departments in Spain [9].

It is estimated that in the next 30 years, life expectancy will decrease by 0.8 years due to illnesses and injuries caused by alcohol use [10].

The WHO defines harmful use of alcohol as causing detrimental health and social consequences for the drinker, the people around the drinker and society at large [11]. Given the relevance of this public health problem, in 2010, the WHO adopted a Global Strategy to Reduce the Harmful Use of Alcohol. In 2019, the General Health Assembly extended this Global Strategy to 2030, including harmful use of alcohol as one of the four key risk factors for major non-communicable diseases [12].

Regarding the impact of this consumption on health services, harmful alcohol use accounts for 15–20% of consultations attended in Primary Care (PC). This means that Primary Care Professionals (PCPs) play an important role in addressing the harmful use of alcohol because they can perform health promotion and prevention actions [13] and conduct long-term follow-ups. Early intervention at this level of care is one of the treatments that have proven more feasible and effective [14]. PCPs have revealed deep knowledge and positive attitudes toward interventions aimed at changing the lifestyle of patients [15]. However, in the case of management of risky alcohol use, a lack of training and a low level of preventive practice have been found among PCPs [16,17,18].

There is currently considerable evidence on the effectiveness of communication interventions in decreasing harmful alcohol use in PC settings [19,20]. A short type of intervention is based on Motivational Interviewing (MI), which has demonstrated its effectiveness in addressing some behavioral health problems in PC [21,22]. This type of interview consists of establishing a collaborative patient-centered conversation, whose purpose is to boost the motivation, self-efficacy and commitment of the person with respect to the behavior change [23]. Some of its elements consist of asking open questions, using techniques to ascertain what the patient says, using reflective listening and summarizing techniques [24]. MI has proven to be a useful clinical method that PCPs can learn and use to achieve lifestyle changes among patients [25]. A systematic review published in 2020 concluded that MI is effective in decreasing alcohol use [26]. Another systematic review in 2018 concluded that there is consistent evidence that this style of conversation is effective in reducing the frequency and volume of short-term alcohol use (<4 months) [27]. Finally, it should also be noted that it is cost-effective in treating the use of toxic substances because it achieves similar results to specific treatments and requires less time and resources [28].

Regarding MI training programs aimed at PCPs, they have proven to be effective in improving their skills with this communicative tool, allowing them to implement this model of the PCP–patient relationship in consultations [29,30,31].

However, despite the demonstrated efficacy of MI, we found few studies in the literature in which a specific intervention was conducted in patients with risky alcohol use in PC. The most relevant MI approaches in our environment have been performed in patients with specific health problems other than harmful alcohol use, such as dyslipidemia [25], diabetes [32], physical inactivity [33,34], or in specific groups and not in the general population [35,36]. In addition, very few studies have evaluated the effectiveness of an intervention beyond 6 months, or the evidence has not been sufficiently consistent. Therefore, the objective of our study was to assess the effectiveness of an intervention based on MI performed by PCPs in PC patients with risky alcohol use.

## 2. Materials and Methods

### 2.1. Study Design

**Figure 1:**
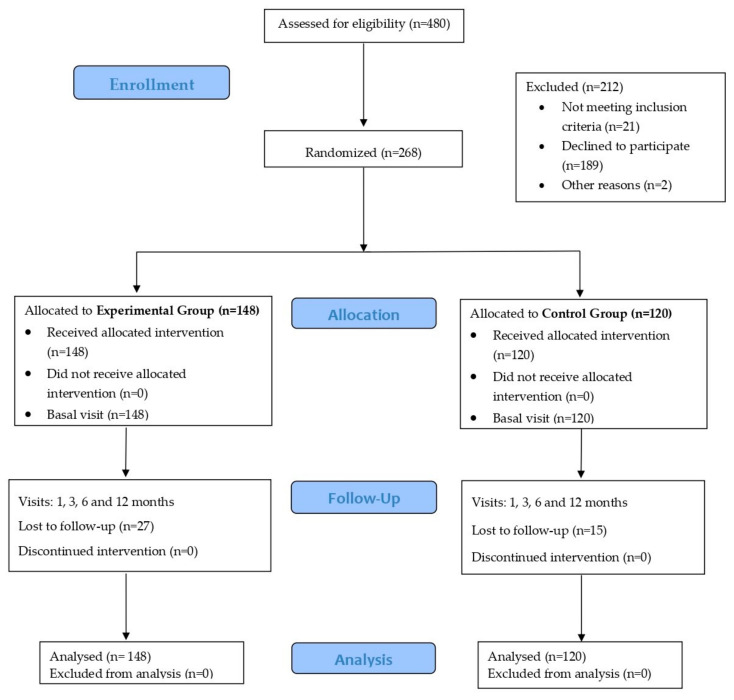
Flow diagram of the study.

This was a multicenter, prospective, two-arm parallel, cluster-randomized, open-label controlled clinical trial with a one-year follow-up period. The study protocol has been previously published [37]. Figure 1 shows the flow chart of the study.

The PCPs were randomized to one of the two study groups:

1. Experimental Group (EG): The professionals implemented an intervention based on MI after receiving training on the management of patients with excessive alcohol use and MI.
2. Control Group (CG): The professionals received training on the management of patients with excessive alcohol use to perform a management based on health advice.

### 2.2. Participants

#### 2.2.1. Study Population

The patients were over 14 years old, with no upper age limit, with risky alcohol use found in PC settings.

#### 2.2.2. Eligibility Criteria

The inclusion criteria for the candidates for participation were as follows: (1) being a PCP (family and community physician, nurse, or family and community medicine or nursing resident); (2) giving consent to participate in the clinical trial. The following exclusion criteria were considered: (1) preliminary competencies self-reported by the professional in MI; (2) refusing to participate in the study.

The inclusion criteria for the patients were as follows: (1) exhibiting risky alcohol use [38,39]: (a) women who consume at least 170 g of alcohol per week (17 Standard Drink Units (SDU)/week); (b) men who drink more than 280 g of alcohol per week (28 SDU/week); (c) patients with a score of 8 or more points in men and 6 or more points in women on the Alcohol Use Disorders Identification Test (AUDIT) [40]; or (d) patients meeting the criterion of “binge drinking” (excessive or intensive use), which is to consume 6 or more SDU for men and 4 or more SDU for women in less than two hours; (2) being 14 years of age or older; (3) consenting to participate in the clinical trial.

The exclusion criteria were as follows: (1) severe cognitive disorder and/or terminal illness; (2) lacking any family support or having no job or means of sustenance; (3) having another severe substance dependence (cocaine, heroin, or designer drugs) for which they are already attending Alcoholics Anonymous or a Drug Treatment Center; (4) any other health issue that, in the professional’s judgment, would prevent the implementation or follow-up of the intervention.

#### 2.2.3. Sample Size

Based on a previous study conducted by members of our team [30], and to detect a 20% difference between the percentage of patients in abstinence (partial or total) between the EG (37%) and the CG (20%), for an alpha error of 5% and a statistical power of 80%, a sample size of 220 subjects (110/group) was calculated. Assuming a loss rate of 5% and with a cluster randomization system being used, the “design effect” was considered. Estimates of the intra-cluster correlation coefficient (ICC) in cluster-controlled clinical trials (CCTs) in PC show that it is generally less than 0.0528. This CCT translates, for a cluster size of 15, into a design effect corresponding to a factor of 1.7. The sample size calculated assuming this value was 394 subjects to recruit (197 in each group).

#### 2.2.4. Recruitment

The recruitment was conducted by field researchers through an active opportunistic search (case finding) of patients recruited by PCPs in their care activities in their health centers.

#### 2.2.5. Random Assignment

The participating professionals were randomized, by simple random assignment, to one of the two comparison groups, stratified according to the center and type of professional. The EPIDAT 4.0 program was used for this randomization

#### 2.2.6. Ethical–Legal Aspects

The research project received the authorization of the Management of the Health District of Cordoba and Guadalquivir and the approval of the Clinical Research Ethics Committee of the Reina Sofia Hospital of Cordoba (8 January 2021; reference: 4930). The study was performed according to Law 14/2007 of 3 July on Biomedical Research and following the precepts included in the Belmont Report and the Declaration of Helsinki for biomedical research. Informed consent that granted voluntary participation of professionals and patients in the study was requested (Arts. 4, 8 and 9 of Law 41/2002; Art. 12 of the Royal Decree (RD) [223/04]). The processing of the personal data of the subjects who participated in the study was in accordance with the provisions of the European Data Protection Regulation and Organic Law 3/2018 on the Protection of Personal Data and the Guarantee of Digital Rights. Informed consent was obtained from all subjects involved in the study.

### 2.3. Interventions

#### 2.3.1. Patients’ Interventions

Patients in the CG received standard care, which consisted of providing advice and information on the need to change the unhealthy habit of excessive alcohol use, advising abstinence or moderation below the risk limit, in accordance with the recommendations of the Program of Preventive Activities and Health Promotion (PAPPS, Spanish acronym) [41], using the traditional health advice based on the informative model. Patients in the EG received MI-based care (the healthcare professionals in this group having been trained for this type of care in the training workshop) [31] combined with the recommendations of the clinical protocol of action of the PAPPS. Both interventions, performed by the healthcare professionals on the patients in their medical quotas, were integrated into the usual PC consultations.

#### 2.3.2. Follow-Up Period

The follow-up period for patients from both groups was 12 months, with the follow-up conducted by the same professional, and the patients received the same number of visits: 5 scheduled face-to-face visits (at baseline and after 1 month, 3 months and 6 months, with a final call after 12 months), with interspersed telephone calls. The duration of each visit was around 10 min, similar to a regular consultation.

#### 2.3.3. Training of Professionals

Professionals from both groups completed a training program before implementing the interventions with patients. Based on previous experience of our research team [30], the training activities conducted to train PCPs on how to act were assessed to verify their effectiveness [31].

In addition, professionals assigned to the EG had to complete, after the end of each initial visit and the 6-month visit, a questionnaire to assess the extent to which they had conducted an MI-based approach. This questionnaire was the MI assessment scale (MIAS, EVEM in Spanish), which was previously validated in Spanish by our research team [42]. The objective of completing this questionnaire was to obtain self-feedback, verify that an intervention focused on MI was performed, and reinforce the professionals’ knowledge, competencies and skills regarding MI.

### 2.4. Variables

#### 2.4.1. Independent Variables

The following variables were measured: age, sex, previous interventions, marital status, educational level, place of residence, weight, height, body mass index (BMI = weight in kilograms/height in meters squared), associated diseases of interest, hygienic–dietary habits (tobacco use, physical activity and coffee use) and opinion of alcohol consumption.

#### 2.4.2. Dependent Variables

The main outcome variables considered to evaluate the effectiveness of the interventions were three: frequency of use (quantified as SDU/week), the total score obtained with the AUDIT questionnaire, and the transtheoretical model of Prochaska and DiClemente [43].

One SDU of alcohol in Spain is equivalent to 10 g of alcohol, which is approximately the average content of a 100 mL glass of wine or cava, a 300 mL glass of beer, or a 30 mL glass of spirits. An average consumption of ≥28 SDU/week in men and ≥17 SDU/week in women is considered risky alcohol use. This variable was measured in each intervention visit (at baseline and after 1, 3, 6 and 12 months).

The AUDIT questionnaire is a validated instrument [40] consisting of 10 questions divided into 3 conceptual domains. The first domain evaluates recent alcohol consumption and consists of 3 questions (frequency of consumption, usual amount and frequency of binge drinking). The second domain assesses dependence symptoms through 3 items (loss of control over use, increased relevance of use and morning use). Finally, the third domain evaluates harmful alcohol use through 4 questions (feeling of guilt after drinking, memory gaps, alcohol-related injuries and the concern of others about drinking). A result of ≥8 points in men or ≥6 points in women is considered indicative of risky alcohol use. This variable was measured at baseline and at the 6-month and 1-year visits. The progression of patients was measured according to Prochaska and DiClemente’s stages-of-change model at baseline visits and at 6 months. Each professional selected the stage of change in which, by their judgment, their patients were in each visit.

### 2.5. Statistical Analysis

An intention-to-treat analysis was performed, and data from the last observation obtained were used for the analysis of subsequent visits to control the effects of withdrawals and dropouts. The data of the analyzed variables were automatically processed in Google Drive, being treated statistically with the program SPSS v. 20.0. A baseline or initial comparability analysis of the groups was performed. Confidence intervals were calculated for 95% (95% CI) for the main study estimators. A bivariate analysis was performed to assess the relationship between the independent variables and the effect of the intervention, in which the chi-square test and means comparison tests for independent samples, such as the Student’s *t*-test or ANOVA, were used (after checking normality using the Kolmogorov–Smirnov test or the Mann–Whitney U or Friedman tests), along with bilateral contrasts (*p* ≤ 0.05). As measures to assess the effect size and impact of the tested intervention, relative risk (RR), absolute risk reduction (ARR) and the number of subjects needed to treat (NNT) were used as estimators. An online calculator (http://evalmedicamento.weebly.com/calcular/1-calculadora-para-las-medidas-del-efecto-de-resultados-en-salud-variables-dicotomicas (accessed on 1 October 2024)) was used. Finally, a multivariate analysis was performed using unconditioned binary logistic regression, considering as dependent variables risky alcohol use measured in SDU/week and the results of the AUDIT test, controlling or adjusting both models according to the presumed predictors or confounding factors, such as the sociodemographic variables. The Hosmer–Lemeshow test was used to test the goodness of fit of the regression models.

## 3. Results

Eighty PCPs reported their willingness to participate in the field work of the study. However, only 69 PCPs were finally involved, and they provided healthcare in 24 PC centers. By opportunistic search, the professionals invited 480 patients to participate in the study, of whom 268 received the interventions planned in the study, 148 being assigned to the EG and 120 to the CG (Figure 1).

### 3.1. Sociodemographic and Clinicopathological Characteristics

The mean age of the participants was 57.44 ± 12.05 (limits: 16–86 years; 95% CI: 55.99–58.89), 87.3% of whom were men and 12.7% of whom were women. Table 1 shows the sociodemographic characteristics of the participants according to the groups to which they were assigned. No significant differences were found between the EG and CG in any of the variables, except for place of residence (*p* = 0.022).

Regarding the clinicopathological characteristics of the patients, the health problems that were most noted were dyslipidemia (42.2%), obesity (35.1%), diabetes mellitus (18.3%), COPD (13.1%), anxiety/depression (12.7%), alcoholic liver disease (10.1%), ischemic heart disease (7.8%), erectile dysfunction (5.2%) and peptic ulcer (5.2%). No statistically significant differences were found between both groups in the prevalence of these conditions. The BMI was 29.11 ± 4.89 (limits: 14.88–47.17; 95% CI: 28.52–29.70); 31.1% of the patients in the EG and 35.5% of the patients in the CG presented obesity (BMI > 30), with no significant differences between the two groups (*p* = 0.650). Significant differences were obtained between the EG and CG in the percentages of patients with alcoholic liver disease (6.1% and 15%, respectively; *p* = 0.016) and dyslipidemia (36.5% vs. 49.2%, respectively; *p* = 0.037) and who were non-smokers (25.7 vs. 13.3%, respectively) or former smokers (22.3% vs. 32.5%, respectively; *p* = 0.022).

### 3.2. MIAS in the Experimental Group

The mean scores obtained with the MIAS scale, self-administered by the professionals participating in the EG, were 44.02 ± 8.68 (median: 45; limits: 20–56; 95% CI: 42.44–45.61) at baseline visits and 45.43 ± 9.56 (median: 48; limits: 12–56; 95% CI: 43.68–47.18) at 6 months.

### 3.3. Prochaska and DiClemente’s Stages of Change

**Figure 2:**
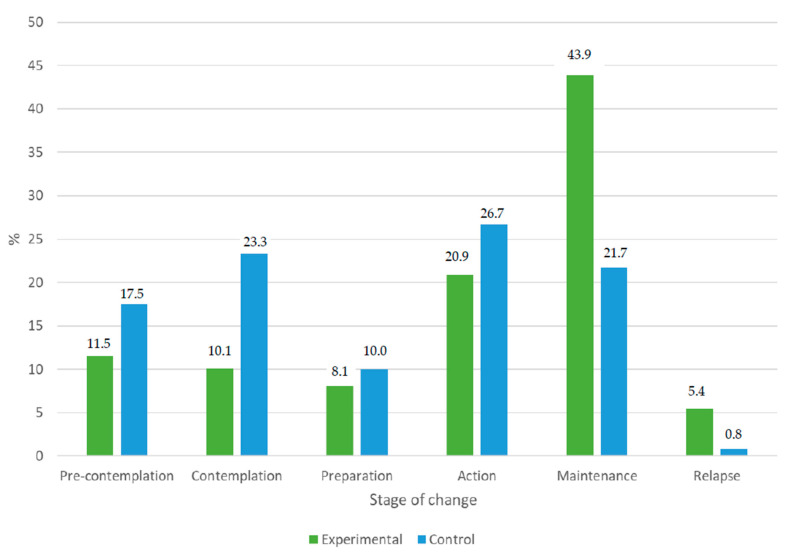
Percentages of participating patients in each of the stages of change according to the Prochaska and DiClemente model, based on the allocation group at 6 months after the intervention.

At the beginning of the intervention, 46.6% of the participants in both groups were in the contemplation stage of Prochaska and DiClemente’s stages-of-change model, 26.9% were in the precontemplation stage, 4.1% were in the action stage and none were in the maintenance stage. At the sixth month of follow-up, the percentage of patients in the contemplation stage decreased to 16.0% and the percentage of those in the precontemplation stage decreased to 14.2%, while the percentage of patients in the action stage increased to 23.5% and the percentage of those in the maintenance stage increased to 34.0%. At 6 months of follow-up, 29.4% of women and 38.5% of men were in the action stage, while 47.1% of women and 32.1% of men were in the maintenance stage (*p* = 0.042). Based on the comparison group, at the beginning of the study, no differences were found in relation to the stages of change (*p* = 0.084), while at 6 months there were statistically significant differences (*p* < 0.001), with 43.9% of the patients in the EG in the maintenance stage, while 21.7% of patients in the CG were. Figure 2 shows the situations of patients in the various stages of change at 6 months after the intervention, based on the assignment group.

### 3.4. Risky Use according to SDU/Week

**Figure 3:**
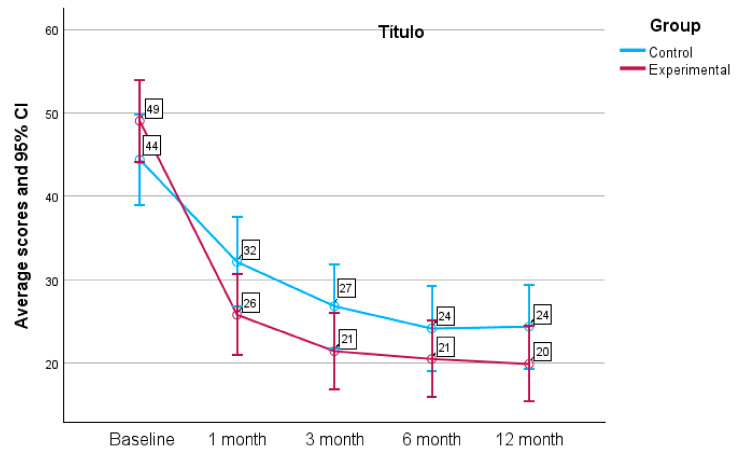
Alcohol use (SDU/week) throughout the study period based on group (95% CI: 95% confidence interval).

**Figure 4:**
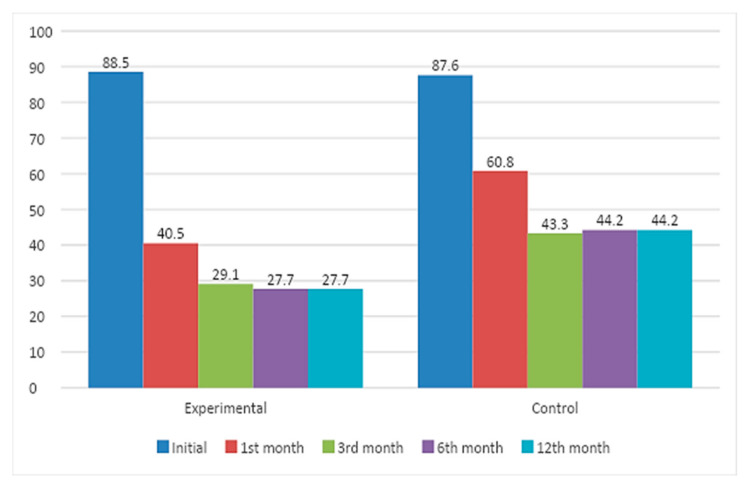
Risky alcohol use based on SDU/week at each follow-up visit in each group (%).

Regarding initial use in terms of SDU/week, the mean in the EG was 49.03 ± 33.91 and 44.42 ± 24.50 in the CG, with no significant differences found between the groups at one month of the intervention, at which point alcohol use had decreased in the EG to 25.81 ± 34.37 SDU/week (95% CI: 20.27–31.35) and in the CG to 32.15 ± 23.02 SDU/week (95% CI: 28.03–36.27), resulting in significant differences (*p* < 0.001). At the six-month visit, weekly SDU use was 20.52 ± 32.67 in the EG (95% CI: 15.21–25.82) and 24.17 ± 21.55 (95% CI: 20.27–28.06) in the CG (*p* = 0.005). Average weekly SDU use at 12 months remained lower in the EG (19.92 ± 31.91 SDU/week; 95% CI: 14.73–25.10) than in the CG (24.39 ± 22.05 SDU/week; 95% CI: 20.41–28.38; *p* = 0.004). Figure 3 shows the weekly alcohol use as SDU values for both groups at each visit. At the baseline visit, it was estimated that risky alcohol use (based on SDU/week) was 88.5% in the EG and 87.5% in the CG, with no significant differences between both groups. At one month of the intervention, risky alcohol use was present in 40.5% of the EG patients and in 60.8% of the CG patients (*p* < 0.001). The visits in which the EG showed a higher decrease in alcohol use were at 6 and 12 months (*p* = 0.005). Figure 4 shows the risky alcohol use, based on the amount consumed in SDU/week at each study visit and according to the comparison group.

Table 2 shows the data on risky alcohol use at each follow-up visit, with measures of the size of the effect of the intervention. The RR at 12 months was 0.63 (95% CI: 0.45–0.87), and the ARR was 16.46% (95% CI: 5.37–27.99), with an NNT of 6 (95% CI: 4–19).

### 3.5. Risky Alcohol Use according to the AUDIT Test Scores

**Figure 5:**
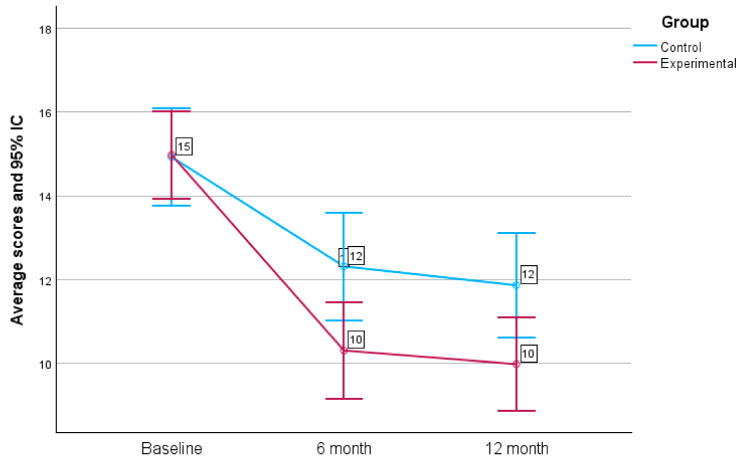
Mean scores obtained in each group with the AUDIT questionnaire at the baseline, at 6 months and at 12 months (95% CI: 95% confidence interval).

At the beginning of the intervention, the mean score for the AUDIT questionnaire was 14.97 ± 6.61 (95% CI: 13.91–16.03) in the EG and 14.93 ± 6.24 (95% CI: 13.81–16.05) in the CG; no significant differences were found between the two groups. At 6 months after the beginning of the intervention, the mean score for the questionnaire was 10.31 ± 7.30 (95% CI: 9.13–11.49) in the EG and 12.32 ± 6.95 (95% CI: 11.07–13.55) in the CG (*p* = 0.014), and it was 9.99 ± 7.10 (95% CI: 8.85–11.13) in the EG and 11.87 ± 6.74 (95% CI: 10.66–13.08) in the CG at 12 months (*p* = 0.016) (Figure 5).

Risky alcohol use was also analyzed according to the scores obtained with the AUDIT questionnaire. The percentage of patients with risky alcohol use at the baseline visit was 95.3% in the EG and 95.8% in the CG, with no significant differences between both groups. Risky alcohol use was present in 27.7% of the EG patients and 44.2% of the CG patients (*p* = 0.057) at the sixth month of the intervention, and it was present in 63.5% of the EG patients and 76.7% of the CG patients (*p* = 0.020) at 12 months. Table 3 presents the data on risky alcohol use based on the AUDIT test in both groups at each intervention visit in which it was measured; the results were obtained with the measures of the size of the effect of the intervention. The RR at 12 months was 0.83 (95% CI: 0.71–0.97), the ARR was 13.15% (95% CI: 2.73%–24.24%) and the NNT was 8 (95% CI: 4–37).

### 3.6. Multivariate Analysis

Table 4 and Table 5 show the effect of the intervention according to the comparison group by multivariate analysis. The odds ratio (OR) of the estimation of risky alcohol use based on SDU/week at 12 months of follow-up according to the allocation group was 0.54 (95% CI: 0.30–0.94), while the OR based on the AUDIT score was 0.36 (95% CI: 0.19–0.66), adjusted according to sociodemographic variables.

There were also differences by sex in the estimation of risky alcohol use based on the AUDIT scores at 12 months of follow-up, estimations being higher in men (OR = 5.30; 95% CI: 2.25–12.47) after adjustment according to the other sociodemographic variables and independently of the assigned group.

## 4. Discussion

The present study represents the first controlled clinical trial published to date to assess the effectiveness, under normal clinical conditions, of a brief MI-based intervention in patients with risky alcohol use recruited at the PC level.

So far, there has been notable evidence of MI for behavioral changes in health in various population groups and settings of the health system [44,45,46]. However, we found variability in the literature regarding the application of MI in the use of addictive substances and its evaluation, which has posed some difficulty for some systematic reviews that have sought to support this type of intervention [47]. Despite this, most of the literature supports the implementation of MI as a type of effective intervention and with solid evidence in relation to alcohol use.

The main objective of the present study was to determine if a brief MI-based intervention was effective in decreasing risky alcohol use in PC patients compared to health advice, which is usually the most common procedure in our environment. Our results show that the MI-based intervention was more effective than the health advice in decreasing the percentage of patients with risky alcohol use. Although in both study groups there was a decrease in alcohol use from the beginning of follow-up, this change was more noticeable in the group that received the MI-based intervention.

The decrease in risky consumption based on the quantification of SDU/week was higher in the EG at 1, 3, 6 and 12 months than in the CG, with statistically significant differences obtained between both groups, the visits at 6 and 12 months showing the highest differences (RR = 0.63). In addition, at the 1-month visit, alcohol use had decreased by almost half in the EG, with an average difference of 6.34 SDU/week between groups. These results support the results obtained in a meta-analysis [48] which concluded that even a single MI session can be effective in improving willingness to change and action aimed at achieving goals of modifying the lifestyle. These data could translate into similar results, for although the traditional brief intervention in PC has been effective in decreasing risky alcohol use [36], the magnitude and speed with which the MI intervention managed to generate a change in behavior was higher since the beginning of the intervention.

As is usual in this type of study, the effect of behavioral interventions in the medium–long term (more than 6 months) is difficult to maintain compared to the values obtained at the end of interventions, and there may even be no differences between groups, as reported by other authors [32,33]. Although the difference in means was lower over time, the results at the beginning of the intervention can also be classified as favorable because the percentage of patients with decreased risky alcohol use remained almost the same at 12 months as at 6 months in both groups. Using MI as a method to facilitate behavior change would further decrease this behavior by up to 16% (ARR: 16.46%) and would decrease the percentage of patients with risky alcohol use (if this is quantified in weekly SDU) by up to 13.5% (according to the AUDIT test) compared to the usual health advice. That is, it would be necessary to perform a motivational approach in six patients with binge drinking (SDU/week; NNT = 6) or eight patients if we consider the AUDIT test score as a reference for one of them to decrease their risky alcohol use (NNT = 8). Another result that provides more consistency regarding the test of the higher effectiveness of the MI intervention compared to the informative advice is the progression of patients according to the stages of change in the transtheoretical model of Prochaska and DiClemente. Thus, at 6 months of follow-up, the percentage of EG patients in the maintenance stage was twice as high as in the CG (43.9% vs. 21.7%, respectively).

In summary, in our study, we obtained a noticeable difference between the group that received the MI intervention and the group that received the standard health advice, with a significant decrease in the percentage of patients with risky alcohol use, this being higher than the decrease among those who received the intervention in the CG, and these results were maintained for up to a year after the start of the study. The intervention was implemented in PC settings, where each user visited the medical room an average of 5.1 times and the nursing room an average of 2.9 times in a year, in contrast to other research studies in which patients with risky alcohol use were recruited at other, more selective care levels, such as hospitals [46]. In addition, the search strategy for candidate patients to be included in the study was active and opportunistic, taking advantage of any PCP–patient encounters, which allowed the systematic screening of alcohol use so that the sample obtained could be quite representative of the population, thus ensuring that the results had greater external validity.

Moreover, one of the problems identified in the literature when evaluating the effectiveness of MI is the inconsistency in the description of interventions and their components [44]. We tried to overcome this issue by using a validated scale for evaluating the elements that must be present in MI (MIAS) [42], which the participants in the EG could use to verify that they were using these elements.

### 4.1. Strengths

One aspect that had to be verified in the study was whether the professionals in the EG performed an MI-based approach. For this purpose, they were asked to check the patient at each visit by filling out the MIAS questionnaire. The results were optimal because the mean score was 44 (out of a maximum of 65 points) at the baseline visit and 45 points at 6 months. If we take as a reference the results achieved by the professionals after the training course they received before starting fieldwork (28.33 points) [31], there was a marked improvement in their skills for managing the MI intervention. Barragan et al. found that the average score on the MIAS was 37.6 after intensive training in MI [28]. Therefore, we can affirm that the professionals in the EG used a motivational approach to a great extent in their interventions.

One of the main strengths of the present study is the high internal validity because it was a multicenter, randomized, controlled clinical trial. Another point to note is the high participation of PC professionals (n = 61) with different profiles (physicians and nurses) who performed their care work in various settings (34 healthcare centers, both urban and rural), which favors a high external validity.

Furthermore, having several tools that measured the effect of the intervention (alcohol use in SDU/week, the AUDIT questionnaire and the stages of change of the transtheoretical model) provided higher consistency in the study’s results. In addition, these outcome variables were evaluated at several visits and not only before and after the intervention, which further contributes to demonstrating the maintenance of the effect.

Another noteworthy point in this study is that, prior to the intervention, both groups underwent a training program in the management of patients with risky alcohol use, receiving training in knowledge, attitudes and tools to manage this type of patients. The CG received a workshop on a brief intervention (the classic approach), while the EG received a workshop on MI conducted by an expert. All participants completed a questionnaire on knowledge in dealing with excessive alcohol use. In addition, those who performed the experimental intervention (MI) were videotaped with simulated patients, and their MI skills were evaluated through the MIAS, before and after training. Thus, it was found that knowledge about this health problem increased in both groups and that the participants in the EG had developed skills in MI [31]. This allowed for higher assurance that the patients included in the EG received a truly MI-based intervention. A positive aspect that we can point out after having performed the workshop on MI with the professionals in the EG is that it is feasible to conduct this type of training and that the knowledge and skills acquired are effective when applied in the consulting room. These findings are consistent with those obtained in other studies suggesting that PCPs can effectively implement MI when they receive training and supervision and that integrating MI techniques can have positive effects in the early stages of treatment [45].

### 4.2. Limitations

One of the limitations of this study is that the sample size was below the predetermined size (n = 394 vs. n = 268), mainly due to insufficient motivation of the subjects invited to participate (many of them were in the precontemplation stage). However, this does not seem to have affected the potency of the study because the results obtained were statistically significant and did not cause type II errors or beta errors.

Furthermore, this study began in 2020, when the COVID-19 pandemic resulted in a high increase in the demand for care and a reorientation of PC services to cope with the healthcare collapse, which was very remarkable at this level of care. The consecutive “waves” in which the incidence of COVID-19 increased had to be responded to, and the exhaustion of healthcare professionals meant that the motivation and time they could devote to the recruitment of patients decreased, forcing them not to be able to give this project a higher priority. This setback was mitigated by extending the deadlines of the fieldwork to recruit more participants, but the response from professionals was somewhat limited.

Another limitation is that only 12.7% (n = 34) of the participating patients were women, which reduces the accuracy of the results obtained in this subgroup. Although, regardless of time and age group, alcohol use in Spain is more widespread among men than among women [49], it is plausible to believe that women were more reluctant than men to agree to participate in the study because there are still cultural reasons why excessive alcohol use in women is less socially accepted than in men, which can lead to more reluctance among women to recognize their health problem.

Furthermore, a selection bias may have occurred due to loss to follow-up, for various reasons. Among the reasons for these losses were not going to a consultation at the scheduled appointment (probably due to lack of motivation), withdrawal or dropout due to personal issues, and a change of reference professional or admission hospital. It is also logical to think that those who dropped out of the study were either in the precontemplation stage or had a high degree of alcohol addiction. It is also necessary to consider the differences between and the heterogeneity in the capacity or efficacy of each professional to achieve adherence to the study and help patients achieve the objective of decreasing their alcohol intake (the average number of patients recruited per professional was 4, but with a range of 1 to 19 patients). No differences between the groups were found regarding the percentage of dropouts or withdrawals, suggesting that there was no differential bias. Moreover, the interview at 12 months was performed in 64% of the sample, so a drag of the data obtained in the previous visits was performed to analyze the data. In addition, the patients who continued with the study were probably the most motivated, so we have to interpret the results at a year of follow-up with due caution because the true effect of the intervention could have been overestimated.

A possible information bias should also be noted, specifically a bias of acceptability, complacency, desirability or social rejection—a bias that occurs when subjects who are interviewed tend to systematically alter their responses in the direction they perceive to be good or desirable, while those considered socially undesirable tend to be avoided or minimized because patients may not tell their status regarding their degree of alcohol use with due accuracy, which may have led to an overestimation of the effect of the interventions, although this could have occurred in both groups equally. Finally, a possible Hawthorne effect or bias of the observed could be indicated [50]—a possibility that is intrinsic to this type of experimental study—but which we think was minimized by having the CG also undergo an intervention aimed at decreasing alcohol use, such that, if it existed, this bias would not be differential between both groups. Predictable confusion biases were controlled by multivariate analysis.

## 5. Conclusions

This study represents the first controlled clinical trial to evaluate the effectiveness of a brief intervention based on MI in patients with risky alcohol consumption in PC. The results of this study showed that MI was more effective in generating faster and longer-lasting behavior changes than the usual health advice, with a greater reduction in alcohol consumption in the experimental group (EG) compared to the control group (CG), especially at 6 and 12 months. The effectiveness of MI was more evident from the start of the intervention and was maintained for up to a year, and its application in Primary Care settings ensures greater external validity.

In addition, it has been demonstrated that it is feasible to implement this communication tool at this level and that PCPs can implement it appropriately after a short training program on this practice.

## Figures and Tables

**Table 1:** Sociodemographic characteristics of the participating patients according to group.

Variable	Total (N = 268)		Experimental Group (n = 148)		Control Group (n = 120)		*p*-Value
Age: mean ± SD	57.44 ± 12.05		56.41 ± 12.84		58.41 ± 10.92		0.225 *
Sex: n/%							0.512
Male	234	87.3	131	88.5	103	85.8	
Female	34	12.7	17	11.5	17	14.2	
Study level: n/%							0.127 **
No studies	8	3.0	5	3.4	3	2.5	
Knows writing and reading	33	12.3	25	16.9	8	6.7	
Primary	141	52.6	74	50.0	67	55.8	
Secondary	58	21.6	31	20.9	27	22.5	
University	28	10.4	13	8.8	15	12.5	
Civil status: n/%							0.163
Married	187	69.8	101	68.2	86	71.7	
Divorced	42	15.7	20	13.5	22	18.3	
Single	32	11.9	21	14.2	11	9.2	
Widow	7	2.6	6	4.1	1	0.0	
Place of residence: n/%							0.022 **
Urban	121	45.2	61	41.2	60	50.0	
Semi-urban	58	21.6	44	29.7	14	11.7	
Rural	89	33.2	43	29.1	46	38.3	

SD: standard deviation; * Mann–Whitney U test; ** Pearson’s chi-square test.

**Table 2:** Risky alcohol use and size of the intervention effect at follow-up visits, based on group.

Visit	Risky Use	Group				Chi^2^ *p*-Value	RR (95% CI)	ARR (95% CI)	NNT (95% CI)
		Experimental		Control					
		n	%	n	%				
Baseline	Yes No	131 17	88.5 11.5	105 15	87.5 12.5	0.799	1.01 (0.93 to 1.11)	−1.01% (−8.79 to 7.23)	−99 (14 to −11)
3rd month	Yes No	60 88	40.5 59.5	73 47	60.8 39.2	<0.001	0.67 (0.52 to 0.85)	20.29% (9.08 to 32.32)	5 (3 to 11)
6th month	Yes No	41 107	27.7 72.3	53 67	44.2 55.8	0.005	0.63 (0.45 to 0.87)	16.46% (5.37 to 27.99)	16.46% (5.37 to 27.99)
12th month	Yes No	41 107	27.7 72.3	53 67	44.2 55.8	0.005	0.63 (0.45 to 0.87)	16.46% (5.37 to 27.99)	6 (4 to 19)

RR: relative risk; ARR: absolute risk reduction; NNT: number needed to treat; 95% CI: 95% confidence interval.

**Table 3:** Risky alcohol use measured according to the scores obtained with the AUDIT test and the size of the effect of the intervention in follow-up visits, based on group.

Visit	Risky Use	Group				Chi^2^ *p*-Value	RR (95% CI)	ARR (95% CI)	NNT (95% CI)
		Experimental		Control					
		n	%	n	%				
Baseline	Yes No	141 7	95.3 4.7	115 5	95.8 4.2	0.825	0.99 (0.94 to 1.05)	0.56% (−4.71 to 6.31)	178 (16 to −21)
6th month	Yes No	99 49	66.9 33.1	94 26	78.3 21.7	0.047	0.85 (0.73 to 1.00)	11,13% (0.74 to 22.11)	9 (5 to 135)
12th month	Yes No	97 51	63.5 34.5	92 28	76.7 23.3	0.020	0.83 (0.71 to 0.97)	13.15% (2.73 to 24.24)	8 (4 to 37)

RR: relative risk; ARR: absolute risk reduction; NNT: number needed to treat; 95% CI: 95% confidence interval.

**Table 4:** Effect of the intervention on risky alcohol use at 12 months, measured in SDU/week, by multivariate analysis, adjusted for sociodemographic variables.

Variables	Beta	*p*-Value	OR	95% CI of the OR
Group (Experimental vs. Control)	−0.621	0.031	0.54	0.30–0.94
Age (years)	−0.08	0.540	0.99	0.97–1.02
Sex (men vs. women)	0.426	0.296	1.53	0.69–3.40
Marital status:				
Married (reference category)				
Divorced	−0.637	0.476	0.53	0.09–3.05
Single	−0.518	0.584	0.60	0.09–3.80
Widowed	−0.625	0.527	0.53	0.77–3.71
Educational level:				
Primary (reference category)				
Secondary	−1.468	0.182	0.23	0.37–1.99
University	−1.320	0.251	0.27	0.28–2.55
Place of residence:				
Rural (reference category)				
Semi-urban	−0.368	0.242	0.69	0.37–1.28
Urban	0.097	0.787	1.10	0.54–2.23

Dependent variable: risky alcohol use (SDU/week); OR: odds ratio; 95% CI: 95% confidence interval of the OR; *p* = 0.043; Hosmer–Lemeshow test: *p* = 0.153.

**Table 5:** Effect of the intervention on risky alcohol use (AUDIT score) by multivariate analysis, adjusted for sociodemographic variables.

Variables	Beta	*p*-Value	OR	95% CI of the OR
Group (Experimental vs. Control)	−1.028	0.001	0.36	0.19–0.66
Age (years)	−0.019	0.15	0.98	0.96–1.01
Sex (men vs. women)	1.668	<0.001	5.3	2.25–12.47
Marital status:				
Married (reference category)				
Divorced	−0.26	0.507	0.77	0.36–1.66
Single	0.539	0.296	1.71	0.62–4.71
Widowed	−0.683	0.426	0.5	0.09–2.71
Educational level:				
Primary (reference category)				
Secondary	−0.249	0.577	0.78	0.33–1.87
University	0.146	0.848	1.16	0.26–5.17
Place of residence:				
Rural (reference category)				
Semi-urban	−0.061	0.872	0.94	0.44–1.99
Urban	−0.211	0.499	0.81	0.44–1.49

Dependent variable: risky alcohol use (SDU/week); OR: odds ratio; 95% CI: 95% confidence interval of the OR; *p* = 0.043; Hosmer–Lemeshow test: *p* = 0.153.

## Data Availability

Data are contained within the article.

## Supplementary Materials

The following supporting information can be downloaded at: https://www.mdpi.com/article/10.3390/healthcare12191970/s1, Collaborators of the Group Name.
