# Supplementary material for: Effectiveness of a Motivational Interviewing-Based Intervention in Decreasing Risky Alcohol Use in Primary Care Patients in Spain: A Controlled Clinical Trial
Source: Healthcare (Basel). 2024 Oct 2;12(19):1970. doi: 10.3390/healthcare12191970 (PMC11477329; doi:10.3390/healthcare12191970)
Supplement: Supplementary file 1 [file healthcare-12-01970-s001.zip › healthcare-3188277-supplementary.pdf]

Collaborators of the Group Name

Annex. Collaborative group ALCO-AP20 (estudioalcoap@gmail.com)

Participants	Center
**Alejandro Camacho Franco**	DCCU Córdoba
**Alicia Moscoso Jara**	Peñarroya-Pueblonuevo
**Alicia Valenzuela Gómez**	Occidente Azahara
**Ana Belen Carmona Casado**	IMIBIC
**Ana González de la Rubia**	Villarrubia
**Ana Morilla Roldán**	Pozoblanco
**Ana Roldan Villalobos**	Carlos Castilla del Pino
**Antonia Carmona Priego**	Levante Norte
**Antonia Toledano Medina**	Montoro
**Antonio León Dugo**	Aeropuerto
**Carmen Jurado Porcuna**	Levante Norte
**Carmen Rodríguez Buza**	Carlota
**Carmen Sánchez Aguilar**	Occidente Azahara
**Celia Pérula Jiménez**	Montoro
**Cristina Rojas Prats**	La Carlota
**Cristina Ruiz Rull**	Montoro
**Elena De Rodrigo Tobías**	Almodóvar
**Elena María De Dios González**	Bujalance
**Enrique Martínez Martínez**	Hospital Universitario de Villalba
**Esperanza Romero Rodríguez**	Carlos Castilla del Pino
**Estrella Castro Martín**	Occidente Azahara
**Eva María Sánchez Cañete**	Polígono Guadalquivir
**Fátima Bravo Ábalos**	Hospital Universitario Reina Sofía
**Fernando Jesús González Martínez**	Posadas
**Francisco López Cañas**	El Higuerón
**Gertrudis Montes Redondo**	Santa Rosa
**Helena Cruz Terrón**	Aeropuerto
**Inés Gutiérrez París**	Almodóvar
**Isabel Jabato Moreno**	Aeropuerto
**Jesús González Lama**	Cabra
**Jesús Villar**	Poniente
**José Angel Fernández García**	Villarrubia
**José Tomás Linares**	Sector Sur
**Juan Baleato Gómez**	Villarrubia
**Juan José León Serrano**	Levante Norte
**Juan Marcos Baños**	Villafranca
**Julia Hervás Jerez**	Sector Sur
**Laura Aranda Domínguez**	Sector sur
**Laura Martín Guerra**	Aeropuerto
**Manuel Marín Agredano**	Pozoblanco
**Manuela Urbano Priego**	Occidente Azahara

**Margarita Fernández Poyatos**	Levante norte
**María Angeles Quesada Román**	Lucano
**María Bello Castro**	Montilla
**María Carmen Luna Moreno**	Cordoba
**María Carmen Ocaña Rodríguez**	Baena
**María del Carmen Castillo**	Occidente Azahara
**Maria del Carmen Membiela Jurado**	Sector Sur
**María Dolores López Espejo**	Occidente Azahara
**María Isabel López Estepa**	Aeropuerto
**María Luisa Soria Cabrera**	Villarrubia
**María Luisa Trigueros Guerra**	Occidente Azahara
**María Sierra Henares**	
**María Reyes Martínez Guillén**	Aeropuerto
**Maria Carmen Membiela Jurado**	Luque
**Marina Guijarro Blanco**	Poniente
**Marta Espejo Marín**	Occidente Azahara
**Miguel Muñoz Álamo**	Occidente Azahara
**Miguel Relaño Pedregal**	Villafranca
**Nazaret María Vargas Berni**	Aeropuerto
**Nazaret Morales Delgado**	Poniente
**Raquel Aguilera Muñoz**	Córdoba
**Raquel Gracia Rodríguez**	Bujalance
**Raquel Sauces Carrillo**	Bujalance
**Rocío Luna Cuevas**	Sector Sur
**Rodrigo Ruz Muriel**	Área Sur de Córdoba
**Rodrigo Sebastian Fernández Márquez**	Lucena
**Rosalía Serrano Berni**	Occidente Azahara
**Sharon Stefany Marín González**	Santa Rosa
**Sofía Chico Tierno**	Occidente Azahara
**Tránsito Porras Castro**	Villarrubia
